# Low LH Level on the Day of GnRH Agonist Trigger Is Associated With Reduced Ongoing Pregnancy and Live Birth Rates and Increased Early Miscarriage Rates Following IVF/ICSI Treatment and Fresh Embryo Transfer

**DOI:** 10.3389/fendo.2019.00639

**Published:** 2019-09-18

**Authors:** Abdelhamid Benmachiche, Sebti Benbouhedja, Abdelali Zoghmar, Peter Humaidan

**Affiliations:** ^1^Center for Reproductive Medicine, Clinique Ibn Rochd, Constantine, Algeria; ^2^The Fertility Clinic, Skive Regional Hospital, Skive, Denmark; ^3^Faculty of Health Aarhus University, Aarhus, Denmark

**Keywords:** luteinizing hormone, GnRH agonist trigger, live birth, early miscarriage, fresh embryo transfer

## Abstract

**Objective:** To examine the correlation between serum luteinizing hormone (LH) levels on the day of GnRH agonist (GnRH-a) trigger and reproductive outcomes following *in vitro* fertilization/intracytoplasmic sperm injection (IVF/ICSI) treatment and fresh embryo transfer, and to identify a pre-trigger serum LH threshold which would be compatible with the most optimal cycle outcome.

**Design:** This study is based on data from a previously published randomized controlled trial conducted from 2014 to 2016.

**Patients:** A total of 322 participants were enrolled.

**Setting:** Private IVF center. Intervention(s): GnRH-antagonist-based IVF cycles triggered with GnRH-a. For the purpose of the study, patients were stratified according to preovulatory LH quartiles (Q1-Q4). Main Outcome Measure(s): Ongoing pregnancy rates (OP), live birth rates (LB) and early pregnancy loss (EPL) rates.

**Results:** The results of the present study showed increasing OP as well as LB rates and decreasing EPL rates with increasing pre-trigger serum LH levels (*P* for trend < 0.06, 0.07, and 0.02), respectively. The absolute difference between the highest LH(Q4) and the lowest LH (Q1) group was 13.4%, 12.1%, and 12% in OP, LB, and EPL rates, respectively. In multivariate regression analysis, a pre-trigger serum LH level of 1.60 mIU/ml was identified as a threshold below which reproductive outcomes decreased. The ROC curve values were statistically significant for OP, LB, and EPL; the AUC (95% CI) = [0.57 (0.50–0.63) *P* < 0.04; 0.57 (0.50–0.63) *P* < 0.05, and 0.60 (0.51–0.70) *P* < 0.04], respectively. A significant positive correlation was found on the day of GnRH-a trigger between serum LH, the number of follicles, serum P4, and serum E2, *p* < 0.03; *P* < 0.03; and *P* < 0.001, respectively.

**Conclusion:** Low serum LH levels on the day of GnRH-a trigger is associated with reduced ongoing pregnancy and live birth rates and increased early miscarriage rates. Our findings suggest a lower threshold of serum LH values on the day of GnRH-a trigger necessary to optimize reproductive outcomes in fresh embryo transfer cycles.

**Clinical Trial Registration**: www.ClinicalTrials.gov, Number: 02053779

## Introduction

Luteinizing hormone (LH) is essential for normal folliculogenesis and oocyte maturation in the natural ovulatory menstrual cycle (1). As early as at a follicle size of 6–8 mm, granulosa cell LH receptors are expressed, although at a low level, explaining the importance of LH from the early stage of follicular growth (2). Concomitantly, the pulsatile secretion of LH increases in frequency during the cycle and the mean LH level increases gradually from approximately 4.8 to 8 mIU/ml (3–5). Beyond the upper limit of the above-mentioned range, a surge of endogenous gonadotropins (FSH and LH) induces ovulation (4). Conversely, in stimulated IVF cycles, the use of GnRH antagonist during the late follicular phase in order to prevent the occurrence of a premature LH surge results in LH levels significantly lower as compared to the natural cycle, preventing the occurrence of premature LH surges (6–8). Accordingly, when GnRH agonist (GnRH-a) is used for final oocyte maturation, low LH levels will be present after the initiation of the GnRH antagonist co-treatment (9–11), raising concerns that LH levels may be too low for optimal cycle outcomes particularly when FSH only is used for ovarian stimulation. Further, several studies have shown that the surge of gonadotropins induced by a bolus of GnRH-a is short and low, respectively, in terms of duration and amplitude (12–17), and that has a negative effect on the early luteal phase gonadotropin and steroids profile (18, 19). Others recently, explored the possible impact of the LH level on the day of ovulation trigger when GnRH-a was used for final oocyte maturation. Indeed, it was found that low LH levels on the day of GnRH-a trigger were associated with a low mature oocyte yield (20, 21). However, their impact on the probability of pregnancy is still unknown. The primary objective of the present study was to examine the relationship between serum LH levels on the day of ovulation trigger and the reproductive outcomes in patients triggered with a bolus of GnRH-a followed by a modified luteal phase support (LPS) and fresh embryo transfer. The secondary objective was to identify a pre-trigger serum LH threshold, if appropriate, which would be compatible with the most optimal cycle outcome.

## Materials and Methods

### Study Design

A secondary data analysis evaluating the relationship between serum LH levels on the day of GnRH-a trigger and the reproductive outcomes. Data were obtained from a randomized controlled trial exploring the impact of mid-luteal GnRH agonist administration on reproductive outcomes in GnRH-a triggered cycles (NTC: 02053779) (22).

### Patients

This study included 322 infertile women who underwent ovarian stimulation, GnRH antagonist co-treatment, GnRH-a trigger and *in vitro* fertilization /intracytoplasmic sperm injection (IVF/ICSI) treatment followed by fresh embryo transfer, using a modified luteal phase support (23–25) at the IVF center Ibn Rochd, Constantine, Algeria, between February 2014 and January 2016.

### Blood Samples and Hormone Assays

Serum LH concentrations were measured at the laboratory of the center, Ibn rochd, Constantine, Algeria on the day of ovulation induction for all participants early in the morning. Sera were analyzed immediately using a Vidas kit (BioMerieux, France). All measurements were performed according to the manufacturer's instructions. The detection limit for the VIDAS LH (LH) assay is 0.1 mIU/ml. The Intra and inter assay coefficients of variation were 2.7 and 3.7%, respectively.

### Study Protocol

The reproductive outcomes as well as luteal phase gonadotropin and steroid profiles of this study have previously been published (22). In brief, hormonal stimulation was performed with GnRH antagonist co-treatment, using recombinant FSH (Puregon., MSD; Gonal F., Merck Serono) for ovarian stimulation. No LH activity was added. Once the leading follicle had reached a size of 13 mm, co-treatment with a GnRH antagonist (Cetrotide. 0.25 mg; Merck Serono) or (Orgalutran. 0.25 mg; MSD) was initiated and continued up until and including the day of induction of ovulation. Ovulation induction was performed with a single bolus of 0.2 mg triptorelin, s.c. (Decapeptyl. 0.1 mg, Ipsen, France) as soon as ≥3 follicles were ≥17 mm in diameter, followed by oocyte pick up (OPU) 36 h later. Retrieved oocytes were fertilized by either IVF or ICSI depending on sperm quality.

### Embryo Transfer and Luteal Phase Support

In alignment with our local embryo transfer policy, one to three embryos were transferred on day 2 or 3 after OPU. A good quality embryo is defined as follows: the number of cells on day 2 is 4 cells and 7–9 cells by day 3, <20% of fragmentation, and regular sized cells.

For luteal phase support, in addition to a bolus of hCG 1,500 IU, IM (Pregnyl.; MSD) given 1 h after OPU, all patients received micronized P (600 mg/day) vaginally (Utrogestan.; Laboratoires Besins-Iscovesco, Paris, France) and estradiol (4 mg/day) orally (Progynova. 2 mg; Schering, Madrid, Spain), beginning on the day after oocyte retrieval and continuing until either a fetal heartbeat was detected by ultrasound examination 5 weeks after OPU or a negative pregnancy test. As part of the study set-up, participants were randomized into two groups, of which the study group received a bolus of Triptorelin 0.1 mg (Decapeptyl. 0.1 mg) 6 days after OPU for additional luteal phase support (22).

### Statistical Analysis

All statistical analyses were performed using the Statistical Package for Social Sciences (SPSS) version 20.0 (SPSS Inc., USA). Descriptive data are presented as mean ± standard deviation or median and range for continuous variables as appropriate, and percentages for categorical variables. Normality was examined by use of the Shapiro–Wilk test. Spearman rank correlation and Mann–Whitney tests were applied when indicated. Non-parametric ANOVA (Kruskal-Wallis test) was used across the four LH quartiles followed by a *post-hoc pairwise* comparison in case of a statistical difference between groups where appropriate. Percentages or rates were compared by use of Pearson chi-square, and Mantel–Haenszel test was computed for trend analysis. The receiver operating characteristic (ROC) curve was defined for serum LH on day of trigger and the area under the curve (AUC) was calculated. Multivariate logistic regression was used to estimate the odds ratio (OR) for the association between LH value on the day of trigger adjusted for all potential confounders and ongoing pregnancy (OP), live birth (LB), and early pregnancy loss (EPL). The LH level on the day of trigger was assessed as quartiles rather than continuous. Variables were included in the logistic regression model if they demonstrated a *P* < 0.03 for the association with outcome in the unadjusted analyses. The model for OP and LB included variables: serum estradiol (E2) levels and serum prolactin levels on day 2, total dose of GnRH antagonist, serum E2, serum progesterone (P4), number of follicles > 11 mm, and serum LH levels on the day of trigger (the first quartile was taken as the reference category), serum LH levels and serum P4 on OPU+7, number of embryos obtained, number of transferred embryos, embryo quality (good vs. bad), and GnRH-a dose on OPU+6 (yes/no). The model for EPL included the following variables: BMI, serum LH levels on the day of trigger (the first quartile was taken as the reference category), serum LH levels and serum FSH levels on OPU+7, the day of embryo transfer (2 or 3), and the GnRH-a dose on OPU+6 (yes/no). All statistical tests were two sided. *P* < 0.05 was considered statistically significant.

## Results

**Figure 1:**
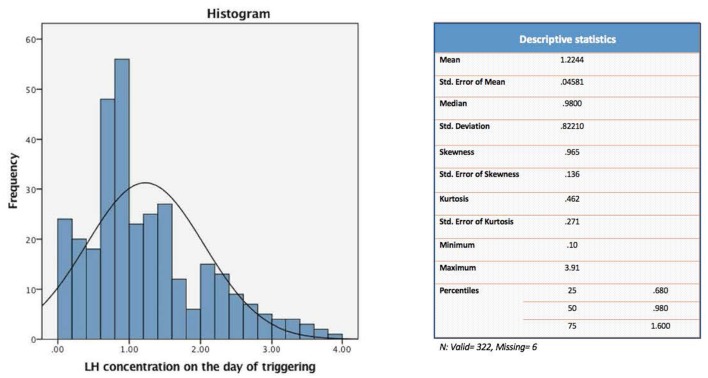
Descriptive analysis of the serum values of LH (mIU/ml) on the day of GnRH-a trigger.

The present study evaluated a total of 322 IVF cycles. Of note, data on preovulatory LH levels were missing in six participants of the original cohort (328 IVF cycles), and hence were dropped from the current analysis. For the purpose of the study, patients were divided into four distinct groups according to their quartile serum LH levels on the day of GnRH-a trigger: [Q1: < 0.68, Q2: 0.68–0.98, Q3: 0.99–1.60, and Q4: > 1.60 mIU/ml] (Figure 1).

Demographic data, stimulation, follicles, oocytes, and embryos.

Baseline characteristics, and stimulation outcomes according to quartiles of serum LH levels on the day of trigger are presented in Table 1. The four groups (Q1, Q2, Q3, Q4) were comparable as regards age, BMI, days of stimulation, total dose of r-FSH, total dose of GnRH antagonist, P4 on day of trigger, number of embryos, and number of transferred embryos. However, significant differences were seen between the highest quartile (Q4) and the lowest quartile (Q1) as regards number of follicles >11 mm and E2 on day of trigger, *P* < 0.04 and *P* < 0.001 respectively.

**Table 1:** Baseline characteristics, and stimulation outcome based on LH levels on the day of GnRH-a trigger.

**Parameter**	**LH Quartile 1** **(<0.68)**	**LH Quartile 2** **(0.68–0.98)**	**LH Quartile 3** **(0.99–1.60)**	**LH Quartile 4** **(> 1.60)**	***P*-value**
Number	83	83	78	78	
Age (years)	31 (23–39)	32 (23–39)	31 (26–39)	31.50 (21–39)	0.99
Body mass index (kg/m2)	27 (18.6–45.8)	27.6 (19.1–40)	28.1 (19.8–37.8)	27 (18.4–43.4)	0.26
Basal LH (mIU/ml)	4.1 (1.3–19.9)	4.8 (1.2–28.2)	4.2 (1.7–17.4)	5.1 (1.9–15)	0.30
No. of days of stimulation	9 (7–14)	9 (6–13)	9 (7–12)	9 (6–15)	0.85
Total dose of r-FSH (IU)	1800 (1400–2700)	1800 (1200–2925)	1800 (1200–2700)	180 (1125–3375)	0.26
Total dose of antagonist (mg)	1 (0.75–1.50)	1 (0.75–1.25)	1 (0.50–1.50)	1 (0.50–1.50)	0.29
No. of follicles on day of trigger	10 a (5–26)	12 (4–30)	15 (4–30)	15 b (4–24)	0.04
E2 on day of trigger (pg/ml)	1611 a (350–6298)	1953 (304–4300)	1916 (426–3000)	2229 b (536–3000)	0.002
P4 on day of trigger (ng/ml)	0.85 (0.36–2.56)	0.98 (0.38–2.63)	0.94 (0.27–2.86)	0.96 (0.38–4.65)	0.08
No. of oocytes retrieved	7 (3–23)	9 (2–30)	8 (3–25)	7 (1–22)	0.45
No. of embryos	5 (1–17)	5 (1–14)	5 (1–16)	4 (1–14)	0.32
No. of embryos transferred	2 (1–3)	2 (1–3)	2 (1–3)	2 (1–3)	0.53

Values are presented as median (range), Groups were compared using Kruskal-Wallis test.

b>a: Statistically significant difference in the post-hoc analysis over the groups Q4 vs. Q1.

*Two-sided P < 0.05 were considered significant*.

### Reproductive Outcomes

**Figure 2:**
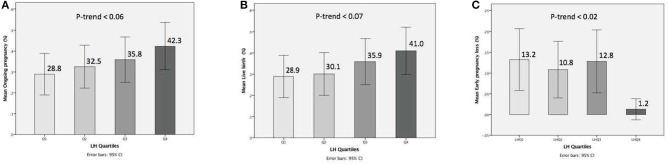
Bar charts represent the ongoing pregnancy **(A)**, the live birth **(B)**, and the early pregnancy loss **(C)** outcomes for LH concentrations when stratified into quartiles. Trend analyzed using Mantel Haenszel test. A trend for increase of ongoing pregnancy, a trend for increase of live birth, and trend for decrease of early pregnancy loss observed with progressively higher concentrations of serum LH (*p*-trend < 0.06) < (*p*-trend < 0.07), and (*p*-trend < 0.02), respectively **(A-C)**. Data are expressed as ongoing pregnancy rates (95% Cl) **(A,B)**, and early pregnancy loss rates (95% CI) **(C)** for each quartile of the serum LH levels.

**Figure 3:**
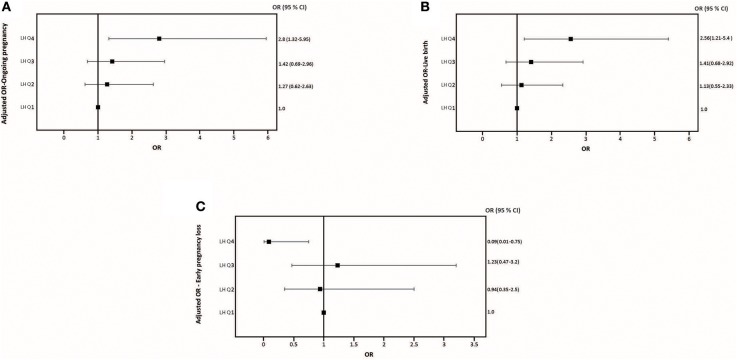
Adjusted Odds ratio (95% CI) for ongoing pregnancy rates **(A)**, adjusted Odds ratio (95% CI) for live birth rates **(B)**, and adjusted Odds ratio (95% CI) for early pregnancy loss **(C)** across quartiles serum LH levels the day of GnRH-a trigger.

The relationship between pre-trigger LH and reproductive outcomes is shown in Figure 2. On one hand, a trend toward increasing OP rates across the lowest to highest quartile of serum LH levels on the day of GnRH-a trigger was seen as the OP rate increased from 28.9% in the Q1 to 42.3% in the Q4 (P for trend < 0.06). Likewise, a trend toward increased LB rates across the lowest to highest quartile of serum LH levels on the day of GnRH-a trigger was seen as the LB rate increased from 28.9% in the Q1 to 41% in the Q4 (P for trend < 0.07). In contrast, a trend toward decreased EPL rates across the lowest quartile (Q1) to the highest quartile (Q4) of serum LH concentration on the day of GnRH-a trigger was seen as the EPL rate decreased from 13.2% in the lowest quartile (Q1) to 1.2 % in the highest quartile (Q4) (P for trend < 0.02). The absolute difference between the highest and the lowest LH groups was 13.4%, 12.1%, and 12% in OP, LB, and EPL rates respectively. The ROC curve values, for OP, LB, and EPL, are shown in Figure S1; the AUC were 0.57, *P* < 0.04; 95% CI (0.50–0.63), 0.57 *P* < 0.05; 95% CI (0.50–0.63) and 0.60, *P* < 0.04; 95% CI (0.51–0.70) respectively. The ROC for EPL outcome has been performed by reversing the dataset labels giving the individuals who got EPL a label of “0” and those who didn't a label of “1”. The difference between these areas and the reference line (area 0.5) was statistically significant for the serum LH measurement (Figure S1). Table 2 summarizes the results of a multivariate regression analysis of the OP rates, LB rates and EPL rates. The results show that in addition to the availability of good embryos for transfer, serum LH level is the most valuable independent predictor of the reproductive outcome. Figure 3 depicts the OR (95% CI) for OP rates, LB rates, and EPL rates according to the quartiles of serum LH (Figures 3A–C), respectively. After adjustment for relevant confounders, OP significantly increased in women with the highest quartile (LH > 1.60 mIU/ml) compared to the lowest quartile Q1 (LH < 0.68 mIU/ml; reference category), OR = 2.80, 95% CI (1.32- 5.95), *p* < 0.007. Figure 3A, LB significantly increased in women with the highest quartile (LH > 1.60 mIU/ml) compared to the lowest quartile Q1 (LH < 0.68 mIU/ml; reference category), OR = 2.56, 95% CI (1.21–5.40), *p* < 0.01. Figure 3B), and EPL significantly decreased in patients with the highest quartile (LH > 1.60 mIU/ml) compared to the lowest quartile Q1 (LH < 0.68 mIU/ml; reference category), OR = 0.09, 95 % CI (0.01–0.75), *p* < 0.02. Figure 3C.

**Table 2:** Multivariate regression analysis of factors related to the cycle outcome.

**Variable**	**Odds ratio (95 % CI)**	***P*-value**
**Ongoing pregnancy**		
Serum LH day of trigger (Q4 vs. Q1)	2.80 (1.32- 5.95)	0.007
Embryo quality (Good vs. Bad)	3.82 (1.68–8.65)	0.001
Embryos (*n*)	1.16 (1.04–1.29)	0.006
Embryos transferred (*n*)	1.59 (1.01–2.50)	0.04
**Live birth**		
Serum LH day of trigger (Q4 vs. Q1)	2.56 (1.21- 5.40)	0.01
Embryo quality (Good vs. Bad)	3.60 (1.60–8.12)	0.002
Embryos (*n*)	1.16 (1.05–1.30)	0.005
Follicles day of trigger (*n*)	0.92 (0.86–0.99)	0.03
**Early pregnancy loss**		
BMI (Kg/m2)	0.91 (0.83–1.00)	0.06
Serum LH day of trigger (Q4 vs. Q1)	0.09 (0.01- 0.75)	0.02

*Serum LH was compared between the first quartile (<0.68 mIU/ml; reference category). and the rest of quartiles (2–4)*.

## Discussion

To our best knowledge, this is the first study to investigate the association between the LH level on the day of GnRH-a trigger and reproductive outcomes in a large cohort of GnRH antagonist co-treated IVF/ICSI treatment cycles. The results of the present study showed increasing OP as well as LB rates and decreasing EPL rates with progressively higher pre-trigger LH levels (P for trend < 0.06; 0.07; 0.02), respectively (Figure 2). After correction for the effect of main confounders, a multivariate regression analysis suggested a serum LH level of 1.60 mIU/ml on the day of GnRH-a trigger as the most appropriate threshold to predict reproductive outcomes (Table 2). Thus, patients with LH> 1.60 mIU/ml exhibited significantly better reproductive outcomes than those with LH < 1.60 mIU/ml (Figure 3). The ROC curve values, though statistically significant for OP, LB, and EPL, did not allow for accurate prediction; the AUC (95% CI) = [0.57 (0.50–0.63) *P* < 0.04; 0.57 (0.50–0.63) *P* < 0.05, and 0.60 (0.51–0.70) *P* < 0.04], respectively (Figure S1). In line with previous reports after human chorionic gonadotropin (hCG) trigger (11, 26–29), the current study using GnRH-a trigger supports the concept that a late follicular phase LH threshold exists below which adverse effects on the reproductive outcomes will occur. Importantly, others previously failed to find any association between LH levels and reproductive outcomes in hCG triggered IVF (30–33). However, studies on the optimal preovulatory LH level in GnRH-a triggered cycles are scarce. Indeed, only two studies showed that low LH level yields a lower number of mature oocytes (20, 21). In contrast, the relationship between pre-trigger LH levels and reproductive outcomes has not been reported before. The area under the curve of LH elicited by a bolus of GnRH-a is significantly less than compared to both the natural cycle and hCG trigger (12–17). Hence, it might be anticipated that low LH levels on the day of GnRH-a trigger might have an even higher impact on assisted reproductive outcomes as compared to hCG trigger. It should be noted that in IVF cycles triggered with hCG, varying cut-off values of LH on the day of trigger have been proposed ranging from 0.5 to 1.2 mIU/ml (11, 26–29), and the majority of them were arbitrarily chosen, and hence not conclusive. In the present study, the threshold of 1.60 mIU/ml suggested by the multivariate regression seems to be slightly higher than the above-mentioned thresholds, assuming that GnRH-a triggered cycles would require a higher LH level to compensate for the inadequacy of the LH activity surge compared to natural as well as hCG triggered cycles. Recently, accumulating evidence has been provided that many potential factors such as GnRH, inhibin, oestradiol, gonadotrophin surging-attenuating factor (GnSAF), and antimüllerian hormone (AMH) may be implicated in the control of circulating LH levels during the follicular phase (34, 35). However, none of these substances fully explain why the LH levels vary from individual to individual. Besides, in antagonist IVF co-treated cycles the circulating LH levels may decrease during the late follicular phase due to the negative feedback of ovarian hormones from multiple follicular developments or after suppressive effect from GnRH antagonist (36). The underlying mechanism by which low pre-trigger LH levels seem to reduce the pregnancy rates has not been fully elucidated. In fact, whether the observed effect of low LH exposure is exerted on the oocyte and/or on the endometrium is not clear. As mentioned, previous studies reported a negative impact of low LH levels on the day of GnRH-a trigger as regards mature oocyte yield. Thus, the study by Meyer el al. (20), showed that a low LH level (LH< 0.5 mIU/ml) on the day of GnRH-a trigger leads to a poor oocyte retrieval. Another recent study (21), reported that patients with a suboptimal hormone response to GnRH-a trigger, as defined by a serum LH< 15 mIU/ml on the morning after GnRH-a administration, had significantly lower LH levels on the day of trigger (1.93 ± 4.65 mIU/ml vs. 2.26 ± 2.25 mIU/ml; *P* < 0.001), and significantly lower mature oocytes retrieved (4.10 ± 5.85 vs. 8.29 ± 6.94; *P* < 0.001) compared to those with adequate response (post-trigger LH> 15 mIU/ml). In contrast, our data failed to find any significant impact of LH levels on the number of mature oocytes which is in agreement with the results reported by Andersen et al. (37) in hCG triggered IVF cycles showing a significant positive association between the late-follicular-phase LH levels and P4 levels, but not the number of oocytes retrieved. Hence, this discrepancy suggests that the impact of low LH levels (LH< 1.60 mIU/ml) may be more relevant to endometrial receptivity rather than to oocyte and/or embryo development. Moreover, our findings are in accordance with a prior study (28) showing that patients with LH levels < 0.5 mIU/ml before the day of hCG trigger in GnRH antagonist cycles exhibited an impairment of their endometrial receptivity since they had decreased implantation rates and LB rates as compared to patients with LH levels > 0.5 mIU/ml, despite significantly higher number of oocytes retrieved and embryos obtained in the group of patients with low LH levels. Interestingly, the same report found that the addition of LH activity in the form of low- dose hCG before ovulation induction significantly enhanced reproductive outcomes in low LH patients. The aforementioned notion is also consistent with a multicenter study (36), which included 333 IVF patients receiving six different doses of the GnRH antagonist, Ganirelix. Administration of the GnRH antagonist started on day 6 of stimulation. In the two highest dose groups, i.e., 1 mg and 2 mg per day, serum LH levels were suppressed well-below 1IU/l on the day of hCG trigger, 0.6 and 0.4 IU/l, respectively. Importantly, despite the fact that the number of retrieved oocytes and the number of good quality embryos were similar to those seen in lower GnRH antagonist dosing groups, implantation rates were significantly lower and early miscarriage rates significantly higher in the 1 mg and 2 mg per day, groups, with no ongoing pregnancies in the 2 mg per day group. Collectively, the above-mentioned effects could be ascribed to lack of up-regulation of endometrial LH receptors. Importantly, endometrial stromal cell apoptosis seems to be reduced by the administration of low dose of hCG (38, 39). Thus, the addition of LH activity in subgroups with markedly suppressed pre-trigger LH levels may have a positive effect on the regulation of the endometrium and hence, implantation (40–43). More studies, including gene-expression analyses, are required in the future to decrypt potential mechanisms involved in the interaction between circulating LH on the day of ovulation induction and the endometrium, particularly when GnRH-a is used for final oocyte maturation.

In the current study, we found that the LH level on the day of trigger is positively correlated with the number of follicles > 11 mm, E2 levels, and P4 levels (*r* = 0.11, *P* < 0.03, *r* = 0.19, *P* < 0.001, *r* = 0.12, *P* < 0.03, respectively) using Spearman rank correlation (data not shown). Thus, our data concur with previous findings, demonstrating the tight correlation between LH and follicular growth (37, 44). To date, several early studies demonstrated contradicting effects of elevated P4 on the day of hCG trigger and reproductive outcomes (45–49). The results of the present study are consistent with the fact that preovulatory P4 levels do not seem to affect reproductive outcomes. Further, the highest OP rate was found in the group of patients who had the highest late-follicular-phase P4 concentrations (i.e., P4 >1.5 ng/ml) 87.5% (98/112) and thus, developed many follicles which is supported recently by Andersen et al. (37). Our results are also in line with two recent reports showing that the possible negative impact of an elevated P4 on the day of hCG trigger seems to be more pronounced in women with low follicle numbers (50, 51). Importantly, the current published data on P4 elevation and IVF outcomes predominantly derive from hCG triggered cycles (52), whereas, there is still a paucity of information addressing this issue in GnRH-a triggered cycles (53). We recognize the limitations of the present study, including the sample size, which prevents statistical detection of further clinically significant differences, the fact that data derive from a *post-hoc* analysis, and the fact that possible circadian variations in LH and progesterone were not taken into account. Moreover, the findings of the current study can not be extrapolated to single fresh blastocyst stage transfer, which is the current mode of modern practice. Finally, the LH assays currently used do not always accurately reflect the LH bioactivity (54).

## Conclusion

This is the first study to assess the impact of low late follicular phase LH levels on reproductive outcomes in GnRH-a triggered IVF cycles. A significant positive correlation was found on the day of ovulation trigger between serum LH quartiles and the number of follicles > 11 mm. Low serum LH levels on the day of GnRH-a trigger is associated with a reduction in reproductive outcomes. Future studies in a larger cohort of patients are needed to corroborate our findings.

## Data Availability

The raw data supporting the conclusion of this manuscript will be made available by the authors, without undue reservation, to any qualified researcher.

## Ethics Statement

This secondary data analysis is based on a previously published randomized controlled trial which was conducted according to the declaration of Helsinki for Medical Research and approved by the Ethics Committee of the University hospital Centre Ibn Badis, Constantine, Algeria. All patients provided written and oral informed consent to participate in the study.

## Author Contributions

AB and PH designed the study, drafted, and edited the manuscript. AB performed data collection, handling of data, and statistical analysis. SB and AZ involved in patient's treatment and review of manuscript. All co-authors accepted the final draft.

### Conflict of Interest Statement

PH received unrestricted research grants from MSD, Merck, and Ferring Pharmaceuticals, as well as honoraria for lectures from MSD, Merck, and Gedeon Richter outside of this work. The remaining authors declare that the research was conducted in the absence of any commercial or financial relationships that could be construed as a potential conflict of interest.
