# Supplementary figures and images for: Low LH Level on the Day of GnRH Agonist Trigger Is Associated With Reduced Ongoing Pregnancy and Live Birth Rates and Increased Early Miscarriage Rates Following IVF/ICSI Treatment and Fresh Embryo Transfer
Source: Front Endocrinol (Lausanne). 2019 Sep 18;10:639. doi: 10.3389/fendo.2019.00639 (PMC6759793; doi:10.3389/fendo.2019.00639)

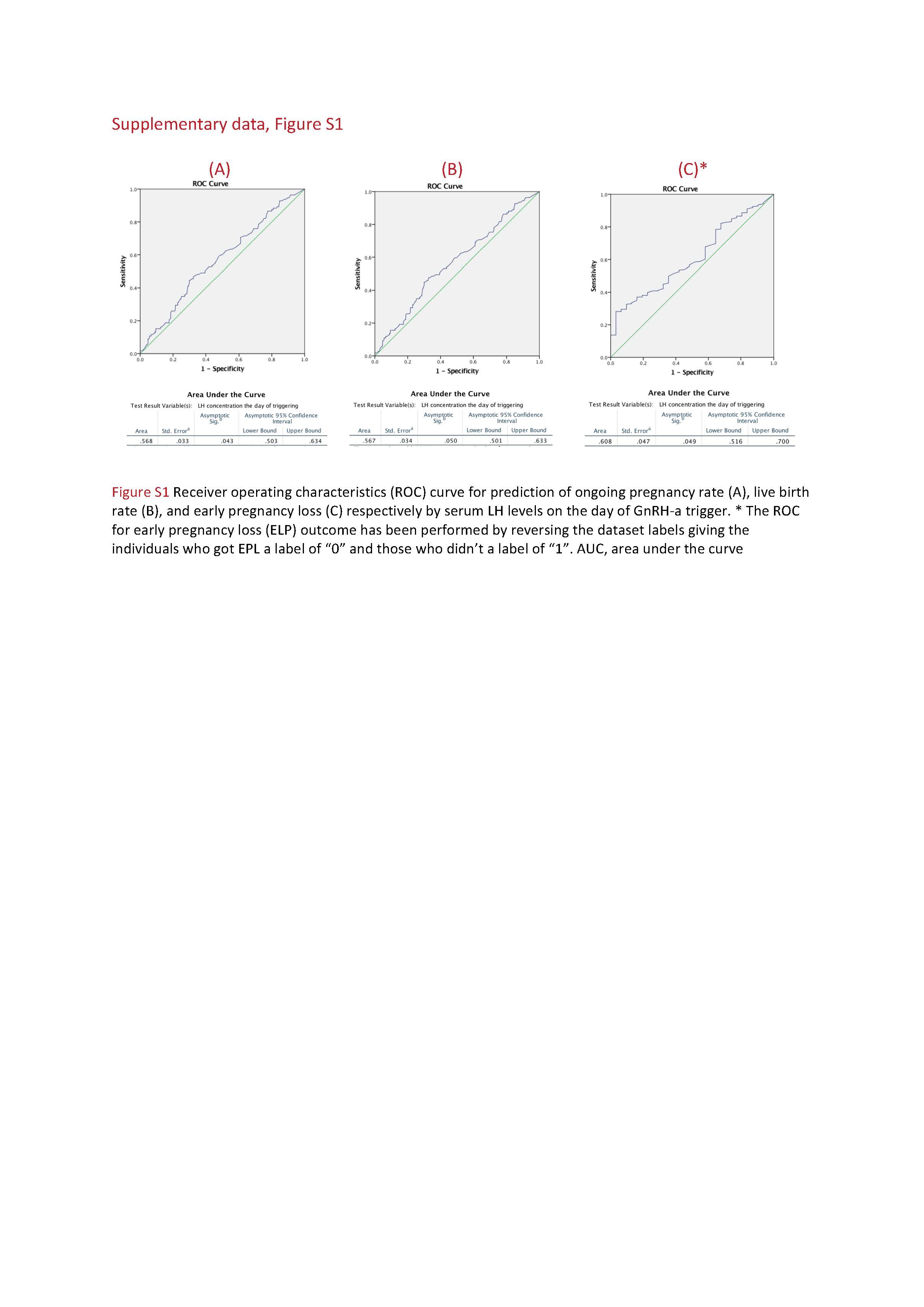

Supplement: Supplementary file 1 [file Image_1.JPEG]
